# New Jersey

**Published:** 1903-05

**Authors:** 


### LEGISLATION.

### NEW JERSEY.

Assembly Bill No. 212 of the New Jersey Legislature, providing for the reopening of the veterinary registry books of the State of New Jersey for three months, was put to sleep in committee in short order by the Legislation Committee of the Veterinary Medical Association of New Jersey.

Assembly Bill No. 125 (amendment to act regulating medical practice) was withdrawn by the introducer on account of objectionable features pointed out by the Veterinary Medical Association of New Jersey and the representatives of other allied professions.

Assembly Bill No. 272 (medical bill), substitute for Assembly 125, was amended in committee at the request of the Veterinary Medical Association of New Jersey, so as to further safeguard the veterinary profession. This bill was favorably reported by the committee with veterinary amendment and finally passed.

There is a provision in the new New Jersey automobile law protecting horsemen. The driver of a motor vehicle is required to stop his machine, when signalled to do so, until the horse or horses are driven past.

